# Seismologically determined bedload flux during the typhoon season

**DOI:** 10.1038/srep08261

**Published:** 2015-02-05

**Authors:** Wei-An Chao, Yih-Min Wu, Li Zhao, Victor C. Tsai, Chi-Hsuan Chen

**Affiliations:** 1 Department of Geosciences, National Taiwan University, Taipei 10617, Taiwan; 2 Institute of Earth Sciences, Academia Sinica, Nankang, Taipei 11529, Taiwan; 3 Seismological Laboratory, California Institute of Technology, Pasadena, CA 91125, USA; 4 Central Geological Survey, MOEA, Taipei 23568, Taiwan

## Abstract

Continuous seismic records near river channels can be used to quantify the energy induced by river sediment transport. During the 2011 typhoon season, we deployed a seismic array along the Chishan River in the mountain area of southern Taiwan, where there is strong variability in water discharge and high sedimentation rates. We observe hysteresis in the high-frequency (5–15 Hz) seismic noise level relative to the associated hydrological parameters. In addition, our seismic noise analysis reveals an asymmetry and a high coherence in noise cross-correlation functions for several station pairs during the typhoon passage, which corresponds to sediment particles and turbulent flows impacting along the riverbed where the river bends sharply. Based on spectral characteristics of the seismic records, we also detected 20 landslide/debris flow events, which we use to estimate the sediment supply. Comparison of sediment flux between seismologically determined bedload and derived suspended load indicates temporal changes in the sediment flux ratio, which imply a complex transition process from the bedload regime to the suspension regime between typhoon passage and off-typhoon periods. Our study demonstrates the possibility of seismologically monitoring river bedload transport, thus providing valuable additional information for studying fluvial bedrock erosion and mountain landscape evolution.

Sediment transport and bedrock incision are critical parameters in the study of landscape evolution^1^,^2^,^3^,^4^,^5^ and are needed for diverse applications including sedimentation engineering, river restoration, and flood hazard mitigation. However, their assessment with traditional geomorphic methods is rarely possible. For example, most measurement techniques (e.g., sediment traps) are *in situ* and cannot be used in extreme flow conditions. Thus, no information is available during typhoon events when sediment is most mobile and erosion processes are strongest.

A potential solution to this data gap is to use remote monitoring by geophones or seismometers that capture the ground vibration caused by surface processes. In the last decade, seismic observations have been widely used to study earth surface processes such as landslides^6^,^7^,^8^, rockslides^9^, rock falls^10^ and debris flows^11^, which we collectively term ‘landquakes’ in this study. Recently, a seismological study has found a correlation between observations of short-period (≤1 sec) seismic noise near rivers and hydrological data, and observed a significant increase in high-frequency seismic energy during the summer monsoon season^12^. Based on seasonal variability in seismic noise levels for a given discharge, termed hysteresis, they suggested that a large portion of this seismic noise is likely due to bedload transport. Other studies similarly observed that the amplitudes of high-frequency seismic noise nearby gravel-rich mountain streams in Taiwan are higher on the rising limb than on the falling limb of each storm event (for the same discharge)^13^,^14^. The aforementioned studies illustrate the potential of using ambient river seismic noise to monitor river bedload transport, which remains difficult with classical approaches. They also point to the need of forward models linking the observed seismic energy to sediment transport^12^,^13^,^14^. Recently, a forward model has been developed to describe the seismic noise induced by sediment transport in rivers^15^, but only a crude inversion for sediment transport was possible. Here, on the other hand, the available hydrological data (e.g., water level) from a river gauge station, geometrical data (e.g., channel-bed cross-section), the sediment grain size distribution, and the river seismic noise observations recorded by our seismic array present an ideal opportunity to investigate the correlation between the bedload transport and river seismic noise level, and to invert for the sediment bedload flux using these seismic observations.

## Results

### Characteristics of the river seismic noise

**Figure 1:**
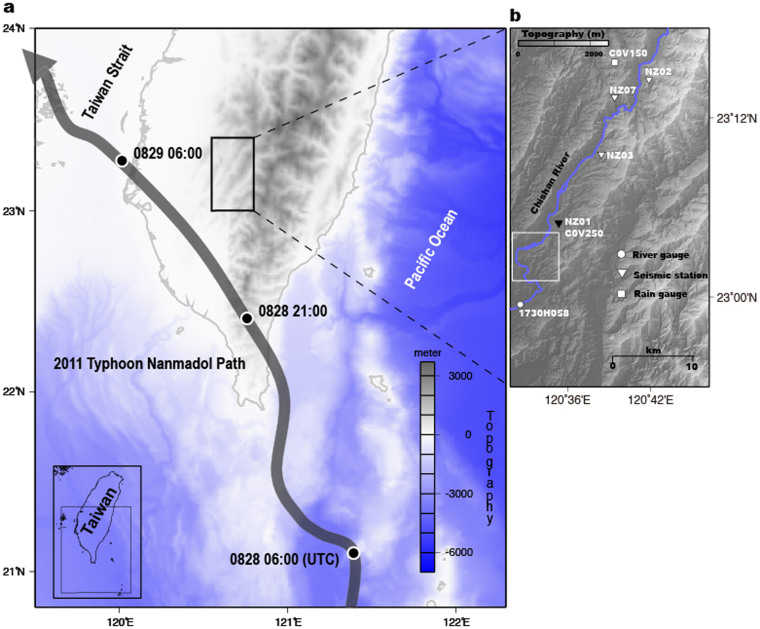
Study area maps. (a) Map of southern Taiwan, with the thick gray arrow depicting the path of Typhoon Nanmadol during 28–29 August 2011. (b) Distribution of the water gauge station (white circle), short-period seismic stations (inverted triangles), and rain gauge stations (rectangles) used in this study. The black inverted triangle shows a co-located seismic station and rain gauge station. The main river is shown by the blue line. Maps are created using GMT (Generic Mapping Tools, http://gmt.soest.hawaii.edu/) software.

**Figure 2:**
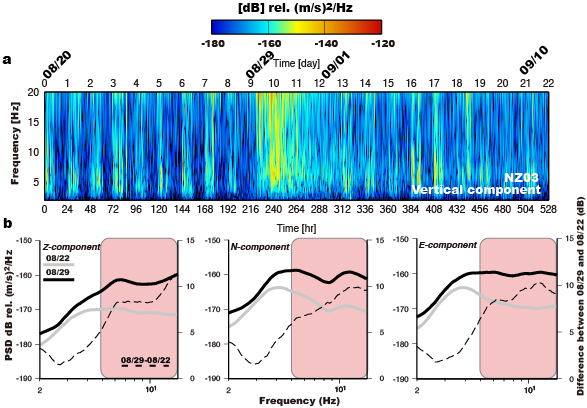
Seismic noise analysis. (a) Spectrogram of the vertical-component continuous seismic record at NZ03. Colors represent the spectrogram power spectral density (PSD) amplitudes in decibels relative to (m/s)^2^/Hz. (b) Mean daily PSDs of the east (left), north (middle) and vertical (right) components for two days (August 22 and 29). The dashed line is the PSD difference between the two days. Pink shaded area in each plot indicates the frequency band 5–15 Hz considered in this study.

To study the characteristics (e.g., frequency range and spatial distribution) of river-generated seismic noise, especially during the passage of Typhoon Nanmadol on 28–29 August 2011, we use records from a short-period seismic array deployed along the Chishan River in the mountain area of southern Taiwan (Figure 1 and see Methods). The Chishan River is a gravel-rich mountain stream, and the drainage area of the Chishan River and the length of its river-channel are 842 km^2^ and 117 km, respectively. Figure S1 shows two examples of one-day continuous records from Station NZ03, located in the vicinity of the river, which reveal large seismic noise amplitudes during the typhoon passage. Moreover, daily variations in the seismic noise are also clear, with larger noise amplitudes during the day (local time 08:00–18:00), reflecting anthropogenic activities (e.g. traffic, excavation, and construction work) in this area. A spectrogram of the vertical-component continuous seismic signal recorded at Station NZ03, with the shortest river-station distance (*r*_0_ = 600 m) of any station, is shown in Figure 2a. The short-period (≤1 sec) seismic signal is particularly well observed during Typhoon Nanmadol (August 29 to September 1). Figure 2b presents one-day average power spectral density (PSD; for further details, see Methods) amplitudes of the three-components during the typhoon passage for the same station NZ03, which are larger by ~5–12 dB relative to those during the pre-typhoon period (dashed line in Figure 2b). These temporal and spectral analyses reveal the existence of high-frequency (5–15 Hz, HF) seismic noise near the river during the typhoon passage, and this HF noise is consistent with previous studies^12^,^13^. Figure S2 shows that there is an increase in the hourly seismic noise level at Station NZ03 from −173 to −152 dB during typhoon passage. In contrast, at Station NZ07, located far from the river (*r*_0_ = 1700 m), the HF signals generated by river processes are not as dominant (Figure S2). These spatial variations in the seismic noise level demonstrate that the river seismic noise has a limited propagation distance for small-magnitude typhoon events due to the rapid decay of the HF signals generated by sediment transport and hydrodynamics (Supplementary S1; Figure S2). These observations provide evidence that the HF noise signals are clearly linked to river processes, so we choose to consider this HF band of 5–15 Hz in our study.

**Figure 3:**
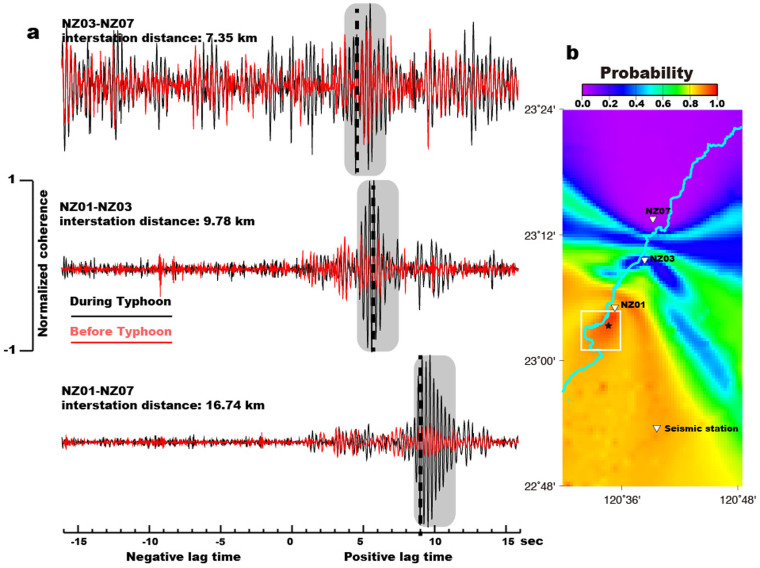
NPCCFs results and source locations of river seismic noise. (a) Stacked NPCCFs for three station pairs NZ03-NZ07 (top), NZ01-NZ03 (middle) and NZ01-NZ07 (bottom) on the day before (red) and during (black) the typhoon period. Gray shaded areas indicate the time windows with highly coherent signals. The dashed lines show the predicted arrival times for the best location (black star in right panel) obtained from back-projection analysis. (b) The shaded colors show a probability map for the locations of noise sources. Red colors denote areas of higher probability which are most probable sources of noise. The Chishan River is shown by a cyan line. The inverted triangles indicate the short-period seismic stations.

To understand the spatial distribution of river seismic noise between off-typhoon periods and during typhoon passage, we use the phase cross-correlation (PCC; ref. 16) function to compute the daily noise cross-correlation function for each pair of receivers in our seismic array (further details in Methods). In Figure S3, we show the vertical-component one-day noise phase cross-correlation function (NPCCF) for the station pair NZ01-NZ03 (9.78 km apart) and observe the emergence of a signal at positive lags between 4 and 8 sec in the NPCCF. This asymmetry of the NPCCF between the acausal and causal parts indicates an inhomogeneous source distribution with a preferential azimuth for the incoming seismic waves. Next, the daily NPCCFs are stacked non-linearly using time-frequency domain phase-weighted stacks^17^, which attenuates signals if they do not appear with a certain regularity and coherence on individual NPCCFs in the pre-typhoon period (August 20 to August 27) and during the typhoon passage (August 28 to September 3). The stacked NPCCFs for each station pair are shown in Figure 3a. The stacked NPCCFs for pre-typhoon period are clearly different from those during typhoon passage, which indicates that strong coherent signals are generated only during the typhoon passage when the Chishan River is under extreme flow conditions (i.e., high transport capacity). Here, we use the back-projection method^18^ (see Methods) to locate the sources of river seismic noise which best explain the stacked NPCCFs for all pairs of stations. In contrast to recent studies which showed that the sources of river seismic noise are concentrated along the steepest portions of rivers^12^,^19^, here we find the source of these highly coherent phases is localized to downstream reaches of the river (white square in Figure 1b and Figure 3b) that have gentler slopes relative to upstream^20^, and instead correspond to high-curvature parts of the river. We propose that the high curvature might cause sediment particle and turbulent flow impacts to be enhanced, in which case we would expect to see a similar asymmetry in the noise cross-correlation functions computed during the off-typhoon period, even though the stream power is not strong. Indeed, the stacked NPCCFs during the pre-typhoon period show strong correlation at positive lags but with less coherency than the results during typhoon passage, especially for the NZ01-NZ03 and NZ01-NZ07 station pairs (red traces in Figure 3a).

### Observed hysteresis

**Figure 4:**
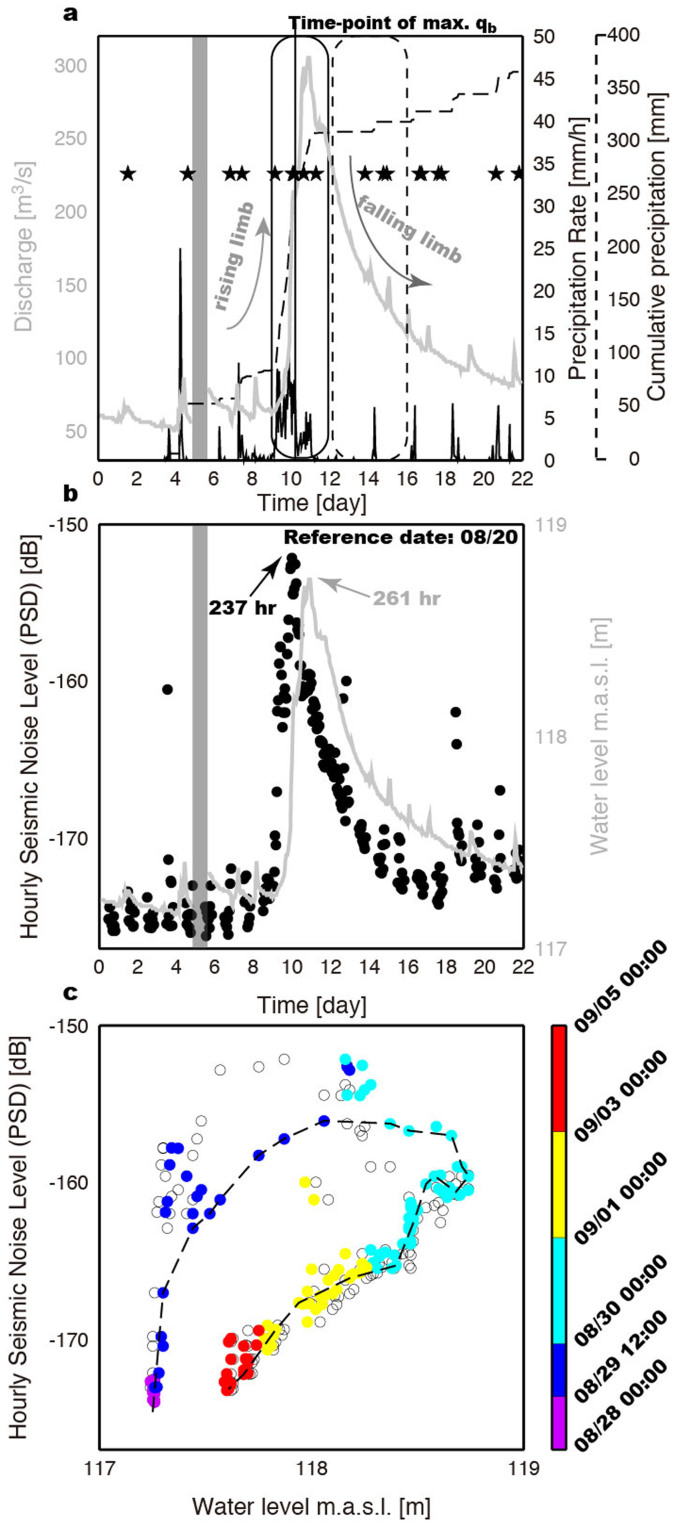
Time-series of precipitation and water discharge, and relationships between river seismic noise and water level. Comparisons of (a) cumulative precipitation (black dashed line), time-series of precipitation rate (black line), water discharge (gray line), and the occurrence of seismologically-detected landquake (hillslope failure) events (solid stars). Solid and dashed rectangles indicate time periods of typhoon passage and off-typhoon period, respectively, which are shown in Figure 5b. The average accumulated rainfall and precipitation rate are calculated from records at two rain gauge stations C0V150 and C0V250 (Figure 1b). The vertical line indicates the time point with maximum *q*_b_; and (b) vertical-component seismic noise PSDs at Station NZ03 and water level in meters above sea level (m.a.s.l.). The hourly PSDs are computed over the frequency band 5–15 Hz after removing contributions from anthropogenic sources (by removing data points during local time 4:00–20:00; gray dots in Figure S2). Water level data in the time interval indicated by the thick gray line is not useable due to recording problems. (c) Comparison of PSD amplitudes with water level at the water gauge station 1730H058 (Shan-Lin Bridge 2). The dashed line is the approximate PSD as a function of water level used in the inversion of bedload flux in this study. The color scale represents the time progression from 28 August to 5 September 2011.

At the river gauge 1730H058 (Shan-Lin Bridge 2; Figure 1b), water level was continuously measured every hour by a stage recorder. However, the suspended sediment concentration, average flow depth, average flood flow velocity, and the channel-bed width were only measured fortnightly. In particular, between 1 July and 30 September 2011, these fluvial measurements were made at an average frequency of four samples per month^20^. As expected, the derived water discharge positively correlates with the average cumulative precipitation (correlation coefficient of 0.95), especially during typhoon passage (Figure 4a). However, we observed strong hysteresis in the HF seismic noise levels relative to the associated hydrological parameters (Figure 4b,c and see section Methods). If turbulent dissipation were the only source of river seismic noise, we would expect a linear scaling between the observed PSD and water level^12^. Although river-induced seismic noise is partly generated by flow turbulence^21^, this mechanism fails to explain the well-developed hysteresis of the observed noise level PSD versus water level. The spatial offset between the seismic and hydrological stations provides another possible explanation for hysteresis. To check whether the spatial offset (of ~25 km in this study) is responsible for the observed hysteresis, we computed the expected temporal lag between the seismic and hydrological data using a field-measured flood flow velocity of 0.5–1.5 m/s (ref. 20). The resulting value of between 4.6 and 13.9 hours is well below the difference between the peak PSD and peak water level (24 hours; Figure 4b), and indicates that most of the hysteresis is not due to the spatial offset between the seismic and river gauge stations. Based on the time difference of the peak PSDs between Stations NZ01 and NZ03 (4 hours; Figure S2) with a spatial offset of 11.4 km, an apparent flow velocity (~0.8 m/s) can be estimated. This seismically estimated value is consistent with field measurements^20^. Thus, in order to invert for the sediment load flux by fitting the observed seismic noise PSDs, we apply a time correction (~6.94 hours) for the spatial offset (~25 km) between the seismic and river gauge stations using the average field-measured flood flow velocity (1.0 m/s). The resulting hysteresis trend (approximated as a dashed line in Figure 4c) is then used in the inversion.

The cause of hysteresis is argued to reflect changes in sediment supply^22^,^23^,^24^. This claim is supported by the known high variability of discharge and high sediment supply during typhoons, and also by the observed high decadal-scale erosion rates of 20–30 mm/year^25^,^26^. To test our claim, we independently estimate the number of hillslope failures (which are expected to lead directly to sediment supply) during our observation time using a seismological method^6^,^27^. This seismic monitoring of hillslopes has the potential to provide a combination of spatial coverage and temporal resolution that is not achievable through conventional methods such as optical remote sensing and *in situ* observations.

This analysis results in a total of 20 detected hillslope failure (or ‘landquake') events (solid stars in Figure 4a; Supplementary S2), and shows that fewer landquakes occur during the falling limb than on the rising limb (Figure 4a). Assuming that material from all seismologically-determined landquakes are delivered into the river, we would predict clockwise hysteresis. Indeed, our results show significant clockwise hysteresis (Figure 4c), indicating that transport capacity was actually high on the rising limb, coinciding with the higher available sediment supply. Thus, a change in sediment supply provides a simple explanation for the observed clear clockwise hysteresis. Other possible contributors to the clockwise hysteresis include time-dependent evolution of riverbed, for example due to grain packing, mobile armoring, or a temporal lag between stage and bedform growth^13^,^14^,^24^,^28^. Due to the lack of detailed data on grain sizes and riverbed evolution in this study, we can only speculate that the observed clockwise hysteresis is mostly due to the changes in sediment supply that are observed.

### Seismologically-determined bedload flux

Here, we adopt a seismic impact forward model which provides an expression for the PSD of the Rayleigh waves generated by the impulsive impacts of saltating particles (ref. 15; further details in Methods). Before inversion, we conducted a set of tests to investigate the influence of model parameters, especially to examine how the grain size of particles (*D*), shortest river-station distance (*r*_0_) and seismic quality factor (*Q*_0_) influence the forward modeling of the PSDs. These tests show that the model prediction for the PSD is strongly dependent on *D* and *Q*_0_ (Supplementary S3). In this study, grain size distribution is calculated using the log-raised cosine distribution, which is analogous to a log-normal distribution except that it includes a cutoff at both large and small grain sizes^15^. In addition, our analysis also shows that the channel-bed angle (*θ*) can significantly influence the transport capacity of the fluvial system. With ample sediment supply, the total bedload flux is limited by the river's transport capacity (Figure S5c). Thus, the angle *θ* is also an important model parameter in the inversion of bedload flux.

**Figure 5:**
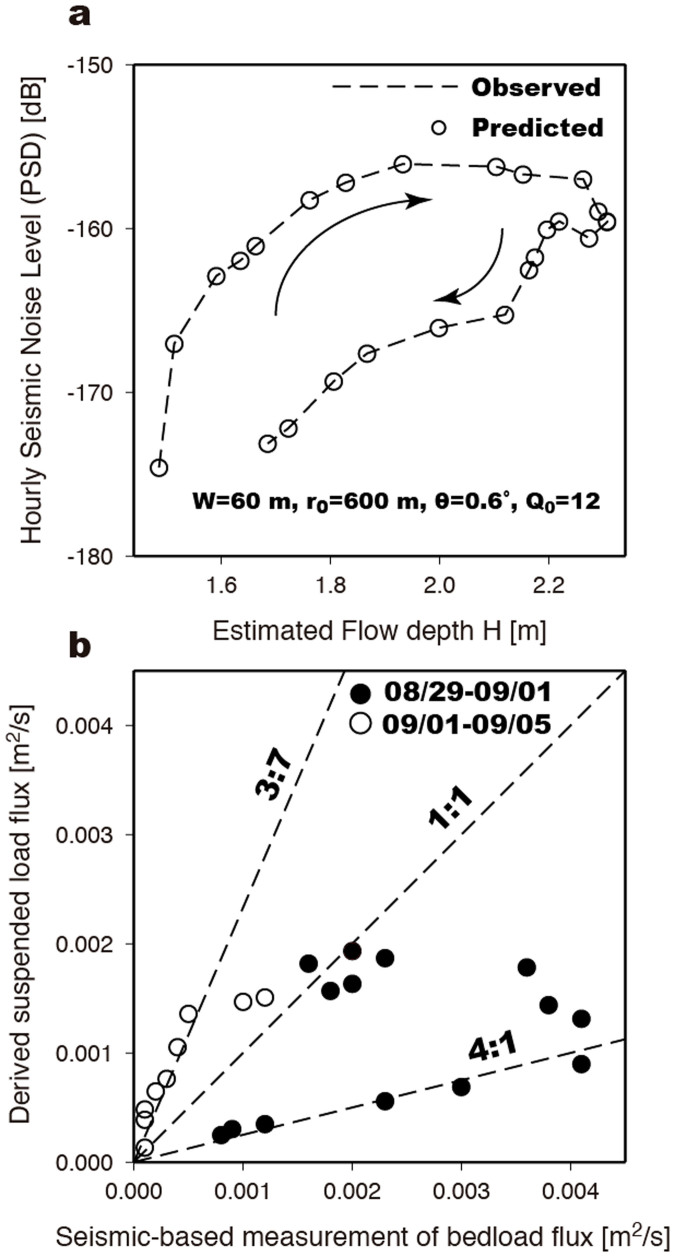
Temporal changes of sediment flux ratio. (a) Results from the inversion of PSD data for bedload flux *q_b_*. The dashed line is the observed PSD for Station NZ03 averaged over 5–15 Hz. The data points are the predicted PSDs as a function of the estimated flow depth. The observed hysteresis is indicated by the black-arrowed curves. (b) The sediment suspended load flux (*q_s_*) derived from the discharge rating curve as a function of the seismologically-determined bedload flux (*q_b_*) obtained by fitting the observed PSD data. Three black dashed lines are shown to indicate given values of the sediment flux ratios of the bedload to suspended load, and are used to aid the discussion of the temporal changes of sediment flux ratio.

Clear hysteresis at Station NZ03 (*r*_0_ = 600 m) in our study, which is pronounced in the HF band with ~9 dB difference amplitude in the rising versus falling limb (Figure 4c), is in rough agreement with previous observations and predictions that show the peak signal due to water noise at lower frequencies^21^,^29^. Thus, we suggest that a large portion of the HF seismic signal is due to bedload transport. However, we note that the excited frequency bands from turbulent flow and bedload sources are significantly influenced by model parameters^21^ (e.g., *r*_0_ and *Q*_0_). If we assume that the full river seismic noise is due to bedload transport, and that the grain size distribution (Supplementary S4.1; Figure S6a) does not change, given the estimates of water flow depth (Supplementary S4.2; Figure S6b), the average channel-bed width (*W* = 60 m; ref. 20), the average channel-bed slope (*θ* = 0.6°; ref. 26), shallow shear-wave speed, and the seismic quality factor (*Q*_0_ = 12; ref. 30), then we can predict the average bedload-induced seismic noise PSD amplitude in the 5–15 Hz range with a given bedload flux. This estimated river seismic noise level can then be used to invert for the total bedload flux. If transport is at capacity, our inversion scheme has enough flexibility in the bedload flux to fit a wide range of PSD observations. Indeed, predicted hysteresis (open circles in Figure 5a) in the seismic noise level PSDs as a function of the estimated water flow depth is in good agreement with the observations. The maximum value of *q*_b_ = 4 × 10^−3^ m^2^/s occurs during the typhoon passage, coinciding with the occurrence of frequent landquake events (Figure 4a). A comparison of the sediment flux of bedload and the derived suspended load is shown in Figure 5b and is discussed in the Discussion.

### Temporal changes of sediment flux ratio

Since continuous monitoring of the suspended sediment load is not always possible, the sediment rating-curve (*Q*_s_ = 1.591*Q*_w_^1.703^, ref. 20), which links the sediment discharge (*Q*_s_, t/d, i.e., in metric tons per day) with water discharge (*Q*_w_, m^3^/s) through a simple power law relationship, has been used to estimate suspended sediment discharge and also suspended sediment flux, *q*_s_. Bedload is sometimes estimated by assuming that it is some proportion of suspended load, but there is much contention in the literature regarding this procedure^31^. While previous studies found that the bedload comprises about 30 ± 28% of the total river load in the high mountains^25^, this fraction is likely to be temporally variable. Therefore, we solve independently for a seismologically-determined bedload flux (*q*_b_) by the inversion of the observed PSD amplitudes, which allows us to investigate temporal changes in the ratio of bedload to suspended sediment flux. Comparing the rating-curve derived *q*_s_ = 2.5 × 10^−4^ m^2^/s during typhoon passage (August 29) with a field-measured *q*_s_ = 2.1 × 10^−4^ m^2^/s (ref. 20) in the pre-typhoon period (August 26), there is only a small difference in the *q*_s_ values, which demonstrates the derived *q*_s_ values are reasonable for further comparison with our inferred *q*_b_ values. Figure 5b shows the comparison between *q*_b_ and *q*_s_ in this study, which is approximately consistent with the ratio 3:7 during the post-typhoon period (September 1 to September 5; dashed rectangle in Figure 4a). In contrast, the bedload to suspended load sediment flux ratio roughly follows a 4:1 trend during typhoon passage (August 29 to September 1; solid rectangle in Figure 4a). In summary, the value of *q*_b_ is generally larger than *q*_s_ during the typhoon passage, and vice versa in the off-typhoon period.

## Discussion

In our results of river seismic noise analysis, the HF noise signals are clearly linked to river processes, and the highly coherent signals in the stacked NPCCFs only appear with positive lag-times during typhoon passage (Figure 3a), which demonstrates the different conditions of transport capacity and sediment flux relative to the off-typhoon period. It also implies that the river seismic noise comes primarily from sources downstream of our study area. Since this downstream reach has a gentler slope relative to upstream, waterfalls or other high-gradient explanations are not likely to explain this observation, and we speculate that the major noise source is mainly from sediment particle and turbulent flow impacting the high-curvature part of river. We also found frequent landquakes occur during the typhoon passage, coinciding with the higher river seismic noise levels, the most intense and prolonged rainfall (rising limb in Figure 4a), and the observed clear clockwise hysteresis (Figure 4c), which can be explained by higher sediment supply. Thus, we suggest that some of the detected landquakes supply the Chishan River with sediment load. However, a more suitable network, consisting of a network of stations surrounding the target river, would improve the spatial resolution and sensitivity of the seismic detections^27^.

In general, the transition from bedload to suspended load is often defined as the point where the bed shear velocity (u_*_) exceeds the particle terminal settling velocity (*w*_s_) (ref. 32). Experimental results indicate that this transition process depends on sediment grain size and transport stage^33^. Thus, we conclude that the discrepancy in the scaling between *q*_b_ and *q*_s_ indicates a complex transition from the bedload regime to the suspension regime between typhoon passage and off-typhoon periods. This discrepancy in the scaling could also result from differences in the amount of hillslope mass wasting into the fluvial system. However, landquakes cannot be captured in the *q*_s_, which is calculated from suspended sediment-rating curve in this study, so we do not have an independent verification of this interpretation. With good constraints on the grain size distribution and the seismic quality factor, our study confirms the potential of using near-river seismic noise observations to estimate bedload flux and to further investigate the temporal changes of sediment flux ratio. This alternative approach to bedload estimation is also useful for studying fluvial bedrock erosion and mountain landscape evolution.

## Methods

### Data

An array of four seismometers was deployed along the main stream of the Chishan River in southern Taiwan. At each site, we excavated a pit about 0.8 m deep and installed the sensor on a level, concrete patch before sealing and covering the instrument. All seismometers are located within 2 km of the river in order to closely monitor river processes. The seismic instruments are equipped with KINKEI KVS-300 short-period velocity sensors with a natural frequency of 2 Hz and 18-bit digital recorders (EDR-7700; http://www.kinkei.co.jp/) and a sampling rate of 100 samples per second. During our monitoring period, one typhoon event, Nanmadol, occurred 28–31 August 2011, and dropped ~306 mm of rainfall at the rain gauge station C0V250 operated by the Central Weather Bureau (CWB) of Taiwan^34^. Data from Station NZ02 was not useable during Typhoon Nanmadol because of power supply problems (Figure 1). To complement our seismic observations, we collected hourly precipitation data recorded at two rain gauge stations C0V150 and C0V250 (ref. 34), both located near the river. Another river gauge station 1730H058 (Shan-Lin Bridge 2), maintained by the Water Resource Agency (WRA) of Taiwan, is 16 km downstream from the seismic station NZ01 (Figure 1b) and records hourly water levels in meters above sea level (m.a.s.l.). Unfortunately, there is a lack of continuous and reliable measurement of water discharge along the Chishan River because of strong hydrodynamics (i.e., the cross section evolves quickly during the storm). The hourly water discharge used in this study was therefore calculated from the rating curve, using a power-law relationship between water level and water discharge^20^.

### Power spectral density (PSD)

Power spectral density (PSD) measurements are used to quantify the seismic background noise in the standard way^35^,^36^. For this analysis, we parse continuous time series at each station into one-day time series sections. Each one-day time series section is divided into 100-s-long segments with 50% overlap, to be efficient in achieving an accurate resolution of the PSDs in the time domain. For each record segment we remove the instrument response, mean and the linear trend before the PSD estimation. For comparison with hydrological and meteorological data, we use an hourly average of the PSD amplitude. In this study, the PSD results are given in decibels [dB] relative to velocity (10 × log_10_[(m/s)^2^/Hz]).

### Phase cross-correlation (PCC)

In general, the ambient noise cross-correlations between two seismic stations can be understood as a tool to detect waves which travel past both stations. The cross-correlation identifies these waves as a function of lag-time, which is the travel-time from one sensor to the other. Thus, the positive or negative lag-time provides information on which direction these waves come from. The PCC is amplitude-unbiased and does not require pre-processing operations such as 1-bit normalization^37^ to remove the influence of energetic features such as earthquakes. Another advantage of PCC is a more efficient signal extraction than the conventional cross-correlation (CCC) scheme, which may enable the use of shorter time windows (days timescale, e.g., typhoon event) to increase the time resolution of monitoring studies. The analytic signal *s*(*t*) of a real time-series *u*(*t*) is uniquely defined as *s*(*t*) = *u*(*t*) + *iH*[*u*(*t*)], where *H*[*u*(*t*)] is the Hilbert transform of the time-series *u*(*t*). Using the exponential form *s*(*t*) = *a*(*t*)exp[*iΦ*(*t*)], we obtain the envelope *a*(*t*) and the instantaneous phase *Φ*(*t*). The PCC is defined as 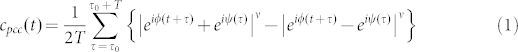
where *c_pcc_* is a coherence functional which measures the similarity of two time-series *u_1_* and *u_2_* as a function of the lag time *t*, and *ϕ*(*τ*) and *ψ*(*τ*) are the instantaneous phases of *u_1_* and *u_2_*, respectively. The PCC is normalized so that |*c_pcc_*| ≤ 1, with *c_pcc_* = 1 indicating perfect correlation and *c_pcc_* = −1 indicating perfect anti-correlation. The sensitivity of *c_pcc_*(*t*) can be increased by setting the exponent ν > 1. We use ν = 1 in this study.

The data processing involves the following steps: (1) Split the continuous vertical-component record at each station into signals of one day in length. (2) Remove the mean and the linear trend, and apply a 2–20 Hz bandpass filter that roughly corresponds to the frequency band of river-induced noise. (3) For each day, compute the noise phase cross-correlation function (NPCCF) between the two time series for each station pair with lag times ranging from −16 to +16 sec. No additional pre-processing is needed for the PCC, but in the conventional cross-correlation (CCC) scheme the data are pre-processed using time-domain and frequency-domain whitening (to make the time-series and spectra of signals more uniform) for each station pair. The aforementioned bandpass filter is applied after spectral whitening. Figure S3 shows a relatively poor signal-to-noise ratio (SNR) in the CCC result, and the high-frequency signals in the CCC result are due to the whitening of the data pre-processing.

### Back-projection method (BPM)

The advantage of BPM is that little prior information is necessary and the results are reasonably stable. In our location procedure, the stacked NPCCFs are bandpass filtered by a fourth-order Butterworth filter with corner frequencies of 2.0 and 6.0 Hz. Next, an envelope of the bandpass filtered records is calculated using a Hilbert transform, and amplitudes are normalized to a maximum value of 1. Previous work concluded that the observed noise cross-correlation functions are dominated by Rayleigh surface waves and/or S waves^19^. We use a recent regional tomography model^38^ in order to minimize the effect of lateral heterogeneities on predicting the S-wave arrival times. The source space is gridded by a mesh with an interval of 0.01° in both latitude and longitude. The source depths are fixed with the free surface topography. Finally, the source locations of seismic noise were determined by a back-projection technique^18^ that maximizes the coherency of the normalized envelope functions among seismic stations.

### Seismic impact forward model

In this study, we adopt the seismic impact forward model of Tsai et al.^15^, which focuses on the seismic energy generated from saltating particles alone while neglecting the particles that are rolling or sliding along the riverbed as well as suspended in the flow and the viscous damping of particle impacts. For a horizontally homogeneous medium, assuming an infinitely long and straight river, an approximate Rayleigh-wave amplitude that decays with depth as *e^−kz^*, with wavenumber *k* and depth *z*, and that all impacts occur randomly in time, the PSD of the Rayleigh-wave velocity time series^15^ (per unit grain size *D* and specific frequency *f*) can be approximated as 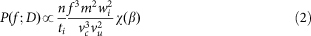
where *n* and *t_i_* are the number of particles with grain size *D* per unit length of river and the average time between consecutive impacts of each particle, respectively. The impact velocity (*w_i_*) of particle of mass *m* is assumed normal to the riverbed. *v_c_* and *v_u_* are the frequency-dependent Rayleigh-wave phase and group velocities, respectively. We use values for the average near-surface shear-wave speed in our study area estimated from borehole seismic data^30^ to fit a power-law shear velocity (*v*_s_) structure as a function of depth (*v_s_* = *v*_0_(*z*/*z*_0_)*^α^*; ref. 39). Based on results of this power-law scaling (*v*_0_ = 2117 m/s, *z*_0_ = 1000 m, and *α* = 0.272), we estimate power-law dependences of *v_c_* and *v_u_* on the frequency. Chi function *χ*(*β*) is geometric factor and *β* is a dimensionless parameter corresponding to the quality factor *Q*_0_. Recent work estimated the P- and S-wave quality factors *Q*_P_ and *Q*_S_ at shallow depths using the CWB borehole array stations and observed both low *Q*_P_ and *Q*_S_ values in our study area^30^. Thus we use an attenuation factor *Q*_0_ = 12 in our modeling.

The rate of particle impact (*n*/*t_i_*) relates to the sediment flux. Based on recent work characterizing these processes in the context of bedrock incision^40^,^41^,^42^,^43^, the expression for *n*/*t_i_* per unit channel-bed length for a given grain size *D* can be summarized as: 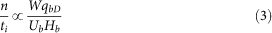
where *W* is the channel-bed width. *U_b_* and *H_b_* are the depth-averaged bedload velocity and bedload layer height, respectively. The quantity *q_bD_* in equation (3) is the volumetric sediment flux per unit grain size *D* per unit channel-bed width *W*, and is determined by the supply of sediment from neighboring hillslopes and from upstream. The total flux 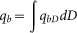, which is a primary fluvial parameter we seek to constrain by the river seismic noise, is limited by the river's transport capacity (*q_bc_*). Details of the aforementioned parameters can be found in Tsai et al.^15^.

## Author Contributions

W.A.C. performed the river seismic noise analysis and the inversion of bedload flux. Y.M.W. and L.Z. helped to co-ordinate the deployment of seismic array. W.A.C. and C.H.C. deployed and maintained the seismic array. V.C.T. assisted in implementing the seismic impact forward model. All of the authors contributed to the data acquisition and interpretation, and the writing of this paper.

## Supplementary Material

Supplementary Information Supplementary Information
