# Supplementary material for: Seismologically determined bedload flux during the typhoon season
Source: Sci Rep. 2015 Feb 5;5:8261. doi: 10.1038/srep08261 (PMC4317699; doi:10.1038/srep08261)
Supplement: Supplementary Information [file srep08261-s1.pdf]

Supplementary Information

Seismologically determined bedload flux during the typhoon season

Wei-An Chao[1*], Yih-Min Wu[1], Li Zhao[2], Victor C. Tsai[3], and Chi-Hsuan Chen[4]

1. Department of Geosciences, National Taiwan University, Taipei 10617, Taiwan.
2. Institute of Earth Sciences, Academia Sinica, Nankang, Taipei 11529, Taiwan.
3. Seismological Laboratory, California Institute of Technology, Pasadena, CA 91125, USA
4. Central Geological Survey, MOEA, Taipei 23568, Taiwan.

This PDF file includes:

S1 Spatial variation of the observed noise level ……..…………..…………..…………2

S2 Seismologically-detected landquake events 2

S3 Dependence of model parameters 5

S4 Estimated parameters before bedload inversion

S4.1 Grain size distribution ……………….…………………………………………..6

S4.2 Water depth ……………………….………………………………………………..7

Supplementary references ……………………………………………………………...7

Supplementary figure captions ………………………………………………………...9

Supplementary figures ……………………………….…………………………………11

S1 Spatial variability of the observed noise levels

Here, we take a look first at daily fluctuations. Figure S2 displays the time series of the seismic noise levels at all available seismic stations. For each station, we calculate the hourly average high-frequency (5-15 Hz, HF) vertical-component PSDs and remove the relatively high seismic noise levels caused by anthropogenic sources (simply cut out the data-points at local time 4:00-20:00; gray dots in Figure S2), with the exception of the time-points during the typhoon passage (corresponding to intense rainfall). As expected, the hourly noise levels during the typhoon passage at Station NZ03 (closest to the Chishan River with shortest river-station distance r_0=600 m) reveal a strong increase in the HF river seismic noise level. This is in contrast to Station NZ01 (r_0=750 m), which is installed on soft sediments in a senior-high school and has the highest noise level. Since NZ07 is located far from the Chishan River (r_0=1700 m), the HF signals generated by the sediment-transport decay rapidly with distance and are not as dominant. These observations provide evidence that the HF noise signals are from river process, and not directly from the typhoon, whose influence would be expected to affect the 3 stations equally.

S2 Seismologically-detected landquake events

Recent studies have established that the seismic signals emitted by landquakes, which can be recorded by local seismic stations, are largely limited to a short-period range (≤ 1 sec,

Supplementary refs 1 and 2). However, this frequency band coincides with those of local earthquakes and anthropogenic sources. To identify different types of seismic events (local/regional and teleseismic earthquakes, and landquakes), we examine the different characteristic spectrograms of these events, as shown in Figure S4. The spectrogram of a landquake typically exhibits triangular-shaped concentration of arriving energy (white dashed line in Figure 4Sa), with an increase over time in high-frequency contents consistent with recent studies (Supplementary refs 1-3). In contrast to landquakes, the spectrograms of local and regional earthquakes show a sharp rise of the seismic energy followed by an exponential decay after the first arrival. Teleseismic events typically exhibit longer durations (a few minutes) and gentler decays of seismic energy than local and regional earthquakes.

In our detection algorithm, we first collect the waveform records from our seismic array and remove the instrument response, mean and linear trend from each trace. The resulting seismograms are then bandpass filtered by a fourth-order Butterworth filter with corner frequencies of 1-5 Hz. Next, we calculate the RMS (root-mean-square) amplitudes of the filtered horizontal component (E-W and N-S) waveforms to create the horizontal envelope functions. Then, we carry out an automated detection of the earthquake and/or landquake activity using a short-term average /long-term average (STA/LTA) approach. Such a detection method is widely used in seismology for the real-time monitoring of seismicity recorded by permanent seismic network. The detection algorithm used in this study employs the ratio

between the average in 0.5 s short and 10 s long time windows in the horizontal envelope functions. STA/LTA ratio threshold of 3 is adopted in this study for triggering the detection on an event. Based on duration and frequency content, we perform a systematic time-frequency analysis to manually identify landquake events with near triangular-shaped spectrograms. Details can be found in Chen et al. (Supplementary ref. 1). In the end, we detected a total of 20 landquake events, which were recorded by at least two seismic stations, with signal durations in the range of 5-33 sec at the closest stations (earliest seismic wave arrival time). The behavior of a landquake is influenced by several factors including geometrical and material properties of the source area. In general, the landquake propagation phase (e.g. fall, slide and flow) is an important factor which controls the seismic signal length. For example, some short-duration events (5-33 sec in this study) are probably characterized by a more prominent free-fall phase with a high slope angle. Details of the discussion can be found in previous work (Supplementary ref. 1).

In addition, we have also analyzed the continuous seismic signals from the Broadband Array in Taiwan for Seismology (BATS, Supplementary ref. 4), and these events cannot be detected at the BATS stations that are farther away since seismic waves from landquakes attenuate quickly. A seismological study found that a landquake, with a collapse area of approximately 0.57 km^2 and a signal duration of 46 sec at the closest station, was recorded at seismic stations up to 40 km away (Supplementary ref. 1). Here, we tentatively attribute our

seismologically-detected landquakes to the relatively small-scale mass-wasting events located in the vicinity of our study area, which likely supplies the Chishan River with the sediment load.

S3 Dependence of model parameters

To explore the dependence of the result from equation (2) (see section Methods in the manuscript) on the fluvial and seismological parameters, we present in Figure S5a the PSD as a function of frequency for two choices of r_0 and three choices of D. We assume that all particles are of the median size $D = D_{50}$, and that $q_b = q_{bD}$ describes the flux of these particles. The results show a sharp increase to peak value at ~5 Hz and a more gradual decrease at higher frequencies. These tests show that the model prediction for the PSD is strongly dependent on sediment grain size (~19 dB increase in the peak PSD amplitude for grain size from D=0.1 m to D=0.25 m at r_0=1000 m). In general, the high-frequency energy decays rapidly with the increase in the shortest river-station distance (r_0). We also examined the dependence on the quality factor Q_0, and found that the value of Q_0 has a significant influence on the PSD amplitudes, especially at frequencies higher than 3 Hz (Figure S5b). In Figure S5c, we plot the PSD as a function of the total bedload flux (q_b) for fixed r_0 = 600 m, H = 2.0 m and f = 5 Hz, but with variable values for D and θ. Results show that θ has only a small influence on the PSD amplitude. In contrast, the river's transport capacity (q_{bc}) is more

sensitive to θ. Based on the aforementioned forward modeling results, good constraints on D and Q_0 are needed for reliable predictions of the PSD.

S4 Estimated parameters before bedload inversion

S4.1 Grain size distribution

We conclude from our forward model tests that the river seismic noise level PSD is strongly dependent on the sediment particle grain size. Thus a good constraint on the grain size distribution is needed for reliable estimates of sediment bedload flux (q_b) using seismic noise observations. Recent work introduced a new log-'raised cosine' distribution of the grain size, which has almost the same shape as a lognormal distribution but has a cut-off at both large and small D (Supplementary ref. 5). Its realistic tail at the high end of the grain size distribution is therefore important for the PSD prediction. The raised cosine distribution is defined by

$$\hat{p}(x;\mu,s) = \frac{1}{2s}[1+\cos\left(\frac{x-\mu}{s}\right)], \quad \text{for } -s < x-\mu < s, \tag{S1}$$

and $\hat{p}(x;\mu,s)=0$ otherwise. $s \equiv \sigma/\sqrt{1/3-2/\pi^2}$, and μ and σ are the mean and standard deviation of the normal distribution, respectively. From the field investigation during our monitoring interval (Supplementary ref. 6), the grain size distribution is approximately 18%, 40%, 22%, 15%, 5% and 0% in bins of 0-2 mm, 2-16 mm, 16-64 mm, 64-256 mm, and > 256 mm, respectively. A best-fit of the log-'raised cosine' distribution to these data yields a

median grain size D_{50} = 0.01 m with a standard deviation σ = 1.75. This best-fitting model results in a grain size distribution of 19.61%, 40.02%, 24.40%, 13.16%, 2.23% and 0% for the same grain size bins. Figure S6a displays the resulting log-'raised cosine' grain size distribution $p(D) \equiv \hat{p}(\log[D];\log(D_{50}),s)/D$.

S4.2 Water depth

In our forward seismic impact model, the measurement of flow depth is needed for the prediction of the river seismic noise PSD amplitude. However, the water flow depth was not continuously measured and only measured at an average frequency of four samples per month from 1 July to 31 September (Supplementary ref. 7). Therefore, we establish here a linear relationship between water level (W_L) and water flow depth (H). The result of the regression, as shown in Figure S6b, has a linear correlation coefficient of 0.94. Using this, we derived the continuous water flow depth from the measured water level as input data.

Supplementary references

1. Chen, C-H., Chao, W-A., Wu, Y-M., Zhao, L., Chen, Y-G., Ho, W-Y., Lin, T-L., Kuo, K-H. & Chang, J-M. A seismological study of landquakes using a real-time broadband seismic network. *Geophys. J. Int.* **194,** 885-898 (2013).
2. Dammeier, F., Moore, J. R., Haslinger, F. & Loew, S. Characterization of alpine

rockslides using statistical analysis of seismic signals. *J. Geophys. Res.* **116,** F04024 (2011).

3. Hibert, C., Mangeney, A., Grandjean, G. & Shapiro, N. M. Slope instabilities in Dolomieu crater, Réunion Island: From seismic signals to rockfall characteristics. *J. Geophys. Res.* **116**, F04032 (2011).
4. Kao, H., Jian, P-R., Ma, K-F., Huang, B-S. & Liu, C-C. Moment-tensor inversion for offshore earthquakes east of Taiwan and their implications to regional collision. *Geophys. Res. Lett.* **25**, 3619-3622 (1998).
5. Tsai, V. C., Minchew, B., Lamb, M. P. & Ampuero, J-P. A physical model for seismic noise generation from sediment transport in rivers. *Geophys. Res. Lett.* **39**, L02404 (2012).
6. Central Geological Survey (CGS). *Geological investigation and database construction for upstream of flood-prone area*. Ministry of Economic Affairs, Taipei ,Taiwan (ROC), pp. 51, 116-131 (2013).
7. Water Resource Agency (WRA). *Hydrological Yearbook of Taiwan*. Ministry of Economic Affairs, Taipei, Taiwan (ROC) (1987-2006, 2010-2011).

Supplementary figure captions

Figure S1. Comparison of one-day three-component seismic velocity records at Station NZ03 before (gray) and during (black) typhoon passage. A daily pattern of seismic noise level can be seen with higher amplitudes during working hours (local time 08:00-18:00), reflecting anthropogenic activities in this area.

Figure S2. Mean hourly PSDs over the frequency band 5-15 Hz for Stations NZ03 (top), NZ01 (middle) and NZ07 (bottom). Arrows indicate the times of the peak PSD values. The 16-hr daily periodicity (local time 04:00-20:00) of high-frequency seismic noise from anthropogenic sources is shown in gray dots. On the other hand, the black dots may be related to the river seismic noise induced by sediment transport. The station names and the shortest river-station distances (r_0) are given in the top-right corner of each plot.

Figure S3. One-day cross-correlograms computed by (top) conventional cross-correlation (CCC) and (bottom) phase cross-correlation (PCC) for station pair NZ01-NZ03.

Figure S4. Comparisons of spectrograms from (a) a landquake event with (b) local, (c) regional and (d) teleseismic (bottom right) earthquakes. White traces are the original vertical-component velocity records. The time-frequency region with the highest energy

arriving from the landquake event is indicated by the white dashed line. The color scale is such that the maximum normalized amplitude is depicted in red while black indicates normalized amplitudes less than 0.5.

Figure S5. (a) PSD as a function of frequency for three different values of D (red: D = 0.05 m; green: D = 0.10 m; and blue: D = 0.25 m) with two different values of the shortest distance r_0 (dashed: r_0 = 600 m and solid r_0 = 1200 m). Values of the other parameters are given in the plot. (b) PSD as a function of frequency for three different values of Q_0 (red: Q_0 = 10; green: Q_0 = 20; and blue: Q_0 = 30). (c) PSD as a function of bed flux for three different values of D (red: D = 0.05 m; green: D = 0.10 m; and blue: D = 0.25 m). Solid and dashed color lines are for $\theta = 1°$ and $\theta = 0.6°$, respectively, while dashed and solid gray lines denote the approximate transport capacity ($q_b = q_{bc}$) for $\theta = 0.6°$ and $\theta = 1°$, respectively. The area where $q_b > q_{bc}$ cannot be achieved.

Figure S6. (a) Log-'raised cosine' grain size probability distribution. (b) Regression for the relationship between the water level W_L and the average flow depth (H). Solid line shows the regression line while the two dashed lines indicate the range of one standard deviation.

Supplementary figures

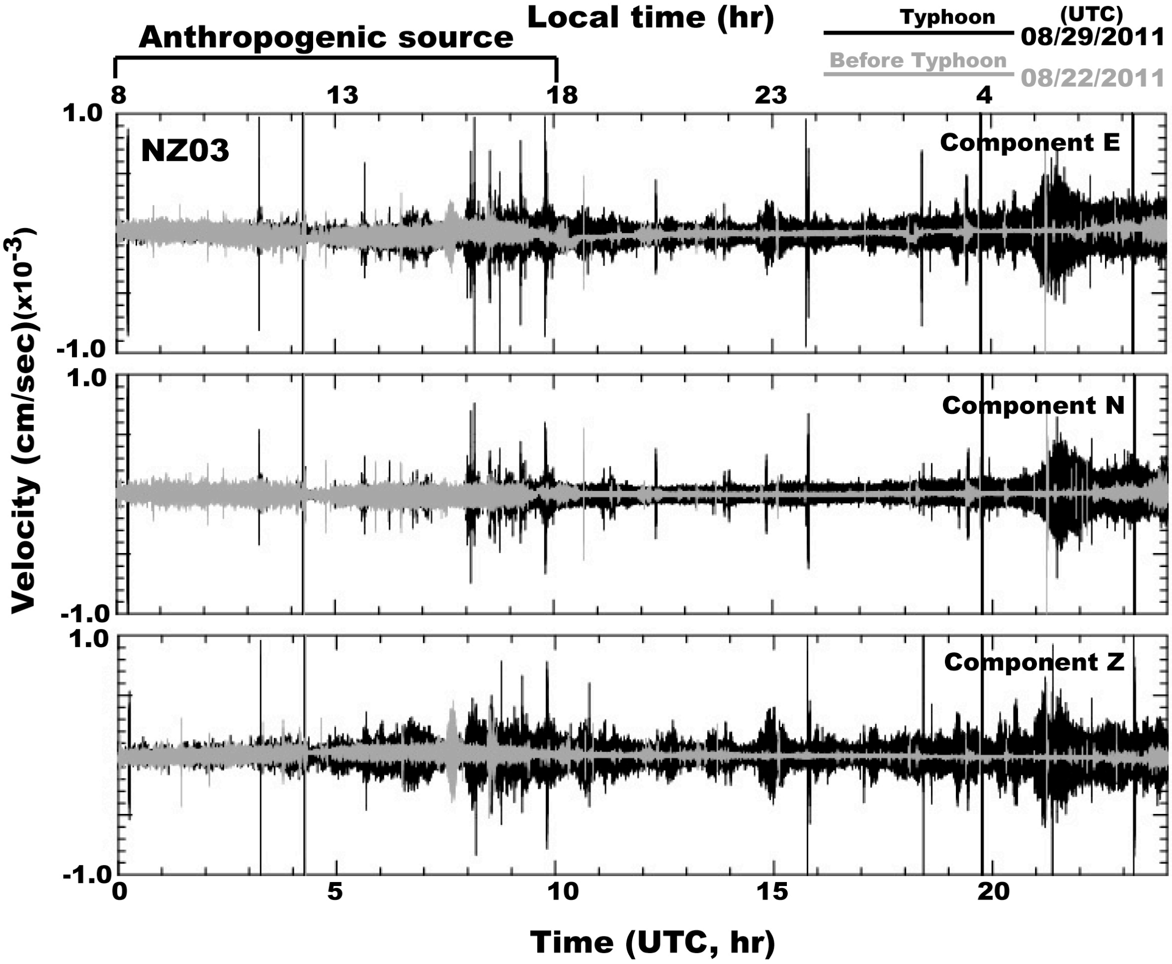

Figure S1

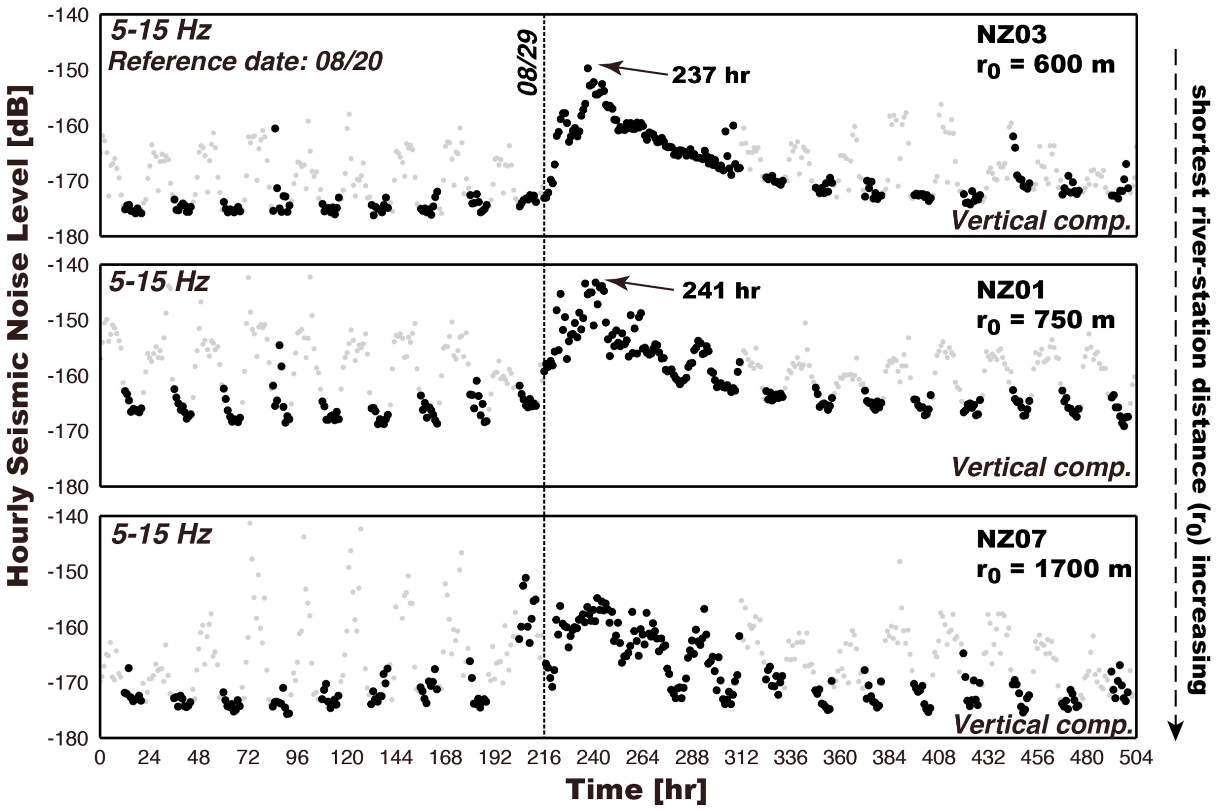

5-15 Hz
Reference date: 08/20
08/29
237 hr
NZ03
r_0 = 600 m
Vertical comp.
5-15 Hz
241 hr
NZ01
r_0 = 750 m
Vertical comp.
5-15 Hz
NZ07
r_0 = 1700 m
Vertical comp.
Hourly Seismic Noise Level [dB]
-140
-150
-160
-170
-180
0 24 48 72 96 120 144 168 192 216 240 264 288 312 336 360 384 408 432 456 480 504
Time [hr]
shortest river-station distance (r_0) increasing

Figure S2

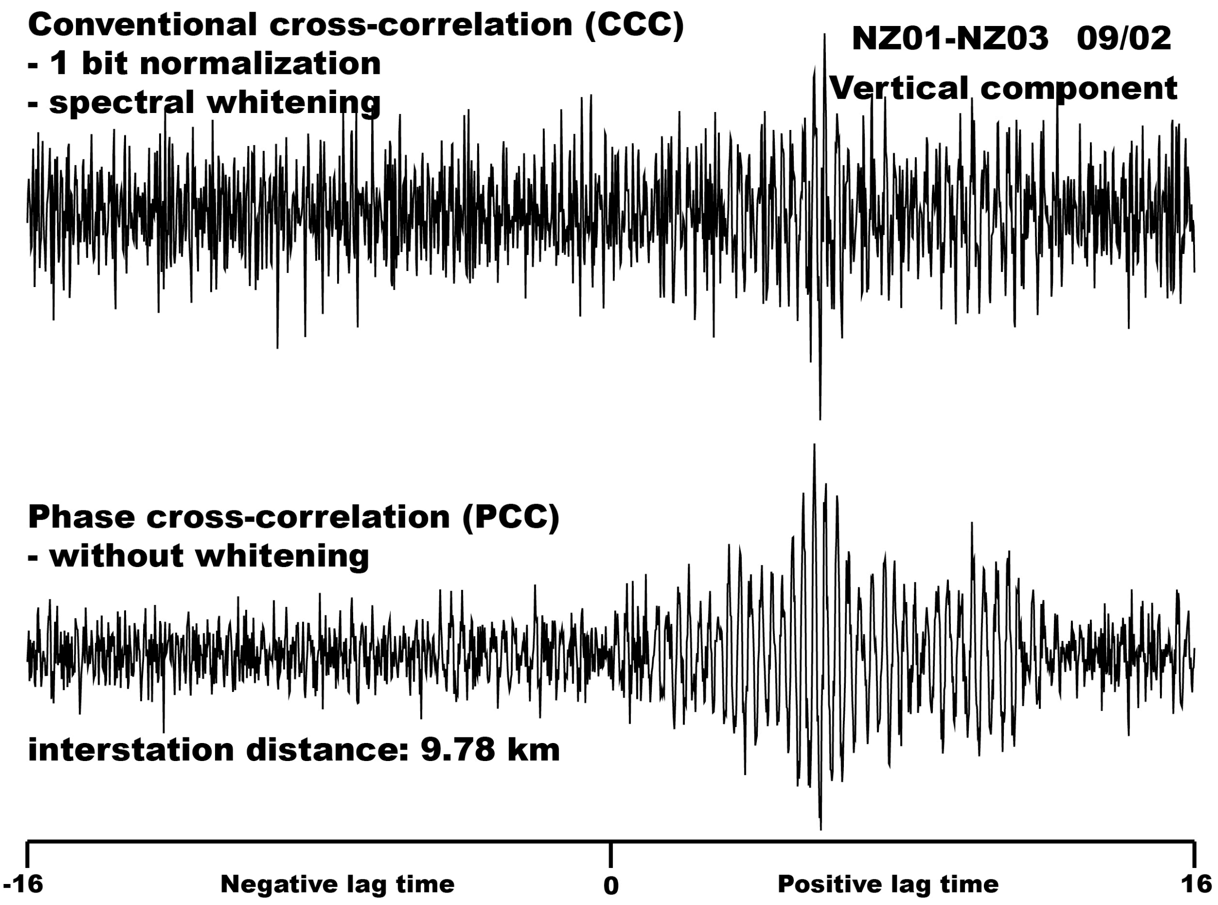

Conventional cross-correlation (CCC)
- 1 bit normalization
- spectral whitening
NZ01-NZ03 09/02
Vertical component
Phase cross-correlation (PCC)
- without whitening
interstation distance: 9.78 km
-16
Negative lag time
0
Positive lag time
16

Figure S3

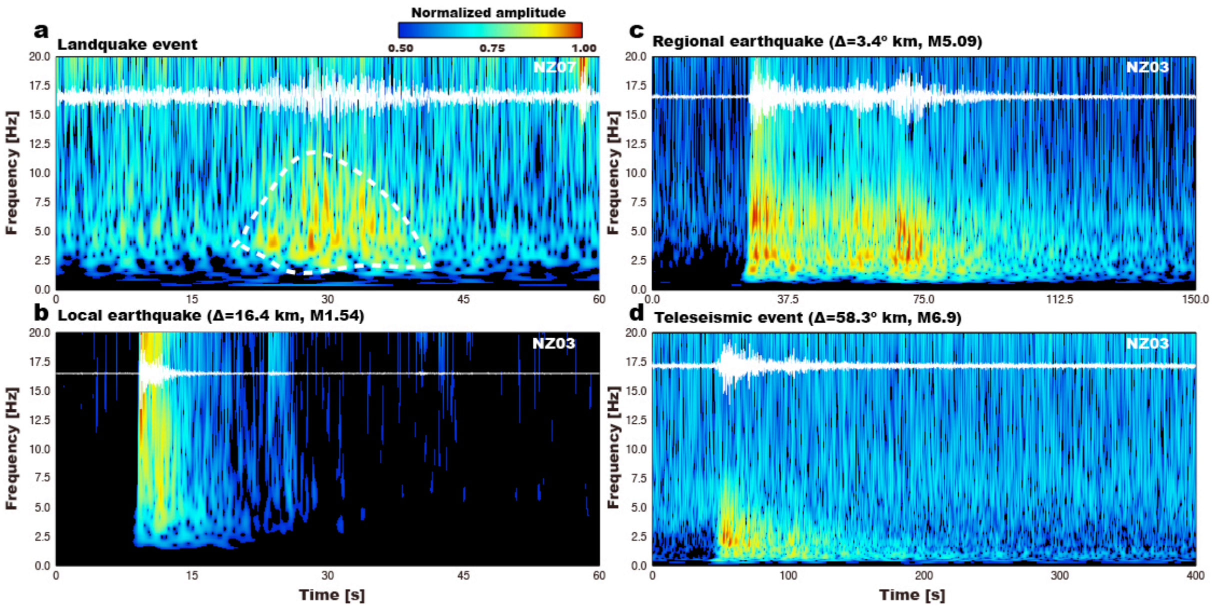
Normalized amplitude
0.50
0.75
1.00
a
Landquake event
NZ07
b
Local earthquake (Δ=16.4 km, M1.54)
NZ03
c
Regional earthquake (Δ=3.4° km, M5.09)
NZ03
d
Teleseismic event (Δ=58.3° km, M6.9)
NZ03
Frequency [Hz]
Time [s]

Figure S4

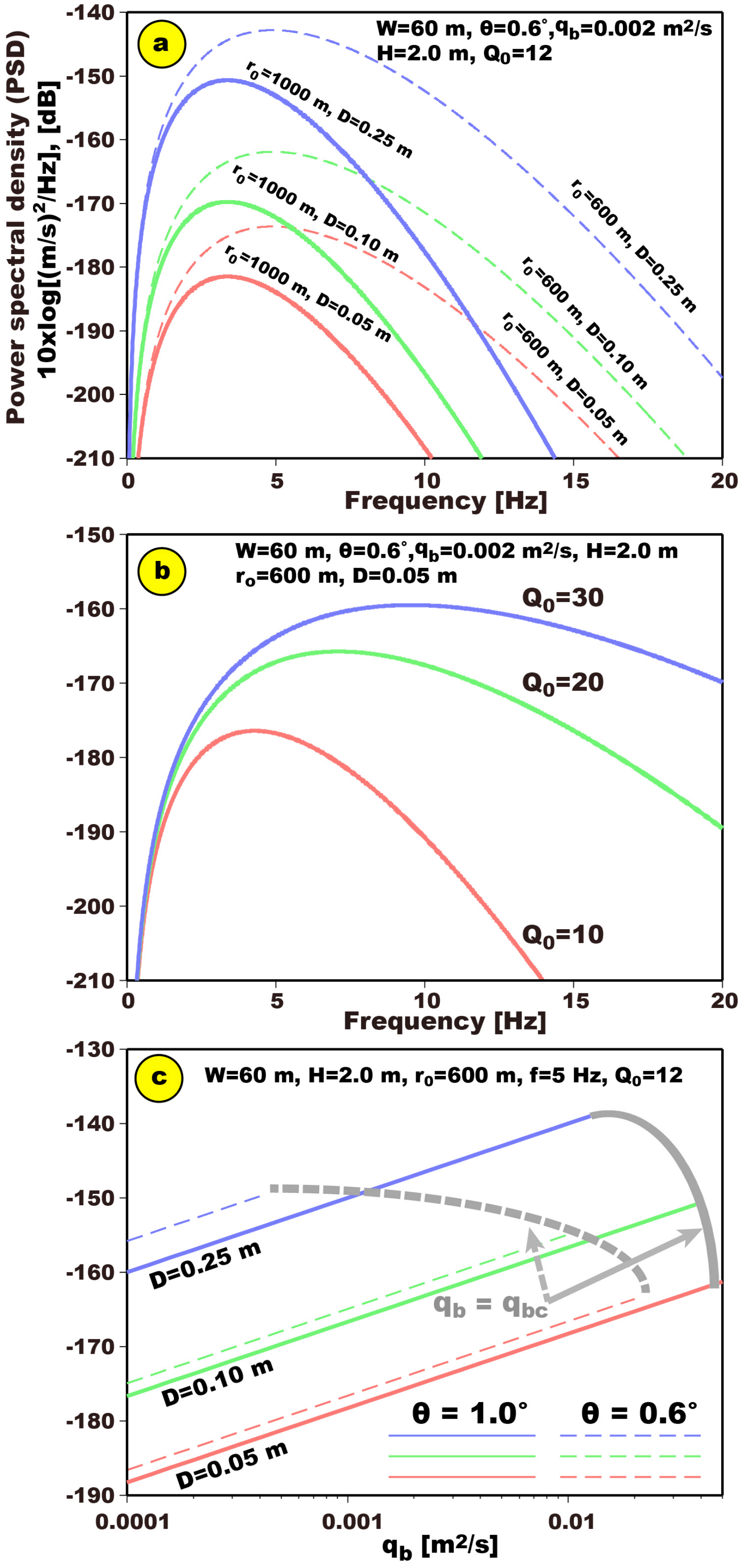

Power spectral density (PSD) 10xlog[(m/s)2/Hz], [dB]
a
W=60 m, θ=0.6°,qb=0.002 m2/s
H=2.0 m, Q0=12
r0=1000 m, D=0.25 m
r0=1000 m, D=0.10 m
r0=1000 m, D=0.05 m
r0=600 m, D=0.25 m
r0=600 m, D=0.10 m
r0=600 m, D=0.05 m
Frequency [Hz]
b
W=60 m, θ=0.6°,qb=0.002 m2/s, H=2.0 m
ro=600 m, D=0.05 m
Q0=30
Q0=20
Q0=10
Frequency [Hz]
c
W=60 m, H=2.0 m, r0=600 m, f=5 Hz, Q0=12
D=0.25 m
D=0.10 m
D=0.05 m
qb = qbc
θ = 1.0°
θ = 0.6°
qb [m2/s]

Figure S5

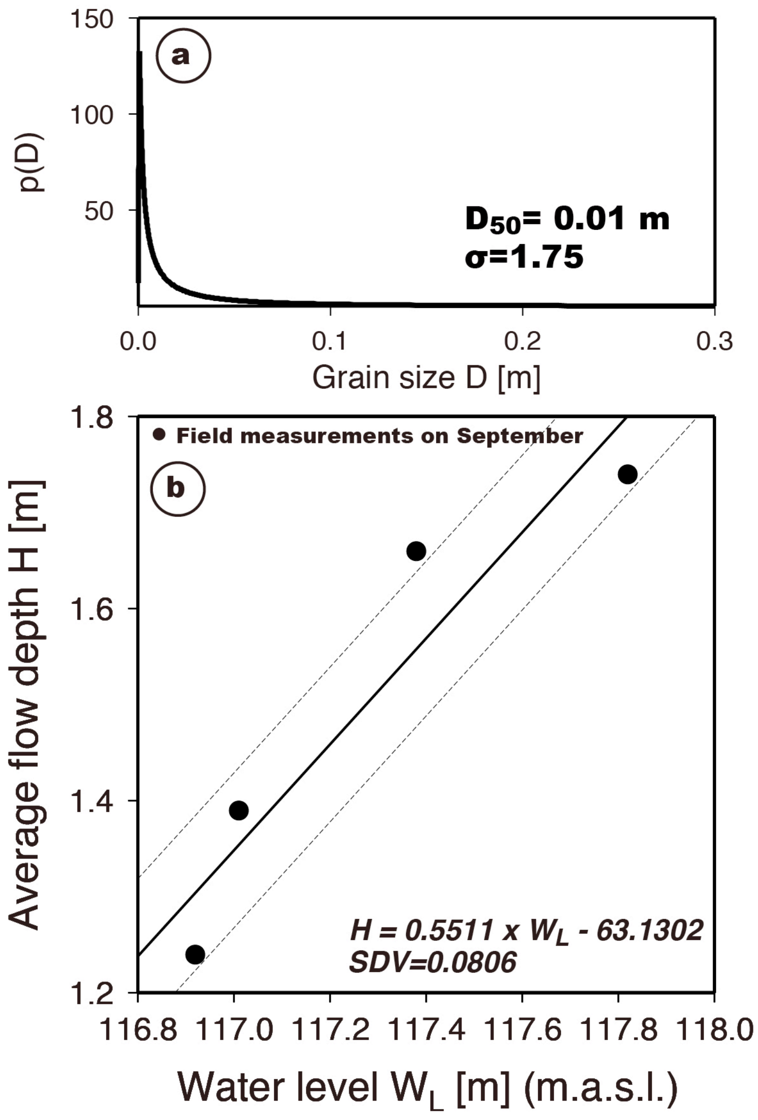
150
100
50
p(D)
a
D50= 0.01 m
σ=1.75
0.0
0.1
0.2
0.3
Grain size D [m]
1.8
1.6
1.4
1.2
Average flow depth H [m]
Field measurements on September
b
H = 0.5511 x WL - 63.1302
SDV=0.0806
116.8
117.0
117.2
117.4
117.6
117.8
118.0
Water level WL [m] (m.a.s.l.)

Figure S6
